# Piezoelectric Thin-Film Actuator for Dynamic Tuning of Micro-Optical Cavities

**DOI:** 10.3390/mi17030345

**Published:** 2026-03-12

**Authors:** Dehua Tan, Pengfei Li, Xuyang Zhou, Qingxiong Xiao, Chaohui Wu, Qixuan Zhu, Miao Lei, Ting Li, Qianbo Lu

**Affiliations:** 1 State Key Laboratory of Flexible Electronics (LOFE), Institute of Flexible Electronics (IFE), Northwestern Polytechnical University, 127 West Youyi Road, Xi’an 710072, China; tandehua@mail.nwpu.edu.cn (D.T.); imleeting@mail.nwpu.edu.cn (T.L.); 2 Shaanxi Key Laboratory of Flexible Electronics, Northwestern Polytechnical University, 127 West Youyi Road, Xi’an 710072, China; 3 School of Marine Science and Technology, Northwestern Polytechnical University, 127 West Youyi Road, Beilin District, Xi’an 710072, China; 4 School of Automation, Northwestern Polytechnical University, 127 West Youyi Road, Beilin District, Xi’an 710072, China

**Keywords:** piezoelectric actuator, F-P cavity, MOEMS, activate modulation

## Abstract

In micro-opto-electro-mechanical systems (MOEMS), the micro-optical cavity plays a pivotal role. As performance requirements for MOEMS devices continue to rise, these cavities must achieve higher performance levels while simultaneously reducing their physical footprint. However, existing high-precision micro-optical cavities face challenges such as high process sensitivity and conflicting trade-offs between dynamic range and precision. To address these issues, piezoelectric thin-film actuators present a viable solution due to their high precision, stroke flexibility, electromagnetic interference resistance, and structural scalability. This study proposes a piezoelectric thin-film actuator based on the $d_{33}$ mode. The device adopts an island-circular structure that integrates a lead zirconate titanate (PZT) piezoelectric film with metal electrodes. By employing particle swarm optimization (PSO) to enhance displacement output and anti-gravity capabilities, the actuator achieves displacement outputs below 100 nm within a compact form factor while maintaining nanometer-level resolution. Simulation and experimental results confirm a first-order natural frequency of approximately 5.8 kHz, along with a reasonable linear displacement response across a 4–6 V drive voltage range. Furthermore, the device demonstrates functionality within a Fabry–Pérot (F-P) microcavity system, enabling active optical path length modulation through precise cavity tuning. This research provides an effective approach to enhancing the dynamic performance and process compatibility of micro-optical cavity devices, advancing the development of next-generation MOEMS systems.

## 1. Introduction

Optical microcavities have widespread applications in optical sensing, integrated light sources, and information communication fields [1,2,3,4,5,6,7]. Particularly within MOEMS, they serve as core sensing units, significantly enhancing device sensitivity and integration levels [8,9,10,11]. However, as device dimensions continue to shrink and performance demands rise, microcavities still face challenges such as performance deviations caused by fabrication tolerances [12,13], trade-offs between dynamic range and stability [14,15], and high sensitivity to environmental fluctuations [16,17]. These limitations constrain their further application in high-precision, high-reliability scenarios.

To improve the performance control and environmental adaptability of micro-optical cavities, research generally incorporates microactuators for actively regulating key parameters such as cavity length and surface curvature [18,19,20,21,22]. Among various microactuators, piezoelectric devices are preferred due to their high precision, rapid response, and ease of integration, making them common in nano-scale driving and positioning applications [23,24,25]. For example, Heo et al. [26] employed a unipolar piezoelectric actuator to drive the tail fin of a biomimetic fish propulsion system; Hall et al. [27] pioneered the application of bipolar piezoelectric actuators in micro-scale flapping-wing robots, enabling centimeter-scale flapping motion for indoor reconnaissance tasks. To further enhance output force, piezoelectric laminated structures or flexible hinge mechanisms are often utilised. Sun et al. [28] developed a piezoelectric microgripper based on a triangular flexible hinge, achieving a maximum claw displacement of 134 µm with a displacement amplification ratio of 15.5. Continually advancing piezoelectric actuators are broadening their applications: Hua et al. [29] designed a precision linear inertial actuator using asymmetric cantilevered bipolar piezoelectric elements, reaching 27 nm resolution, a load capacity of 100 g, and a maximum operating speed of 17 mm/s at 35 Hz. Kim et al. [30] integrated an inchworm structure with a flexible hinge, attaining 8.4 times displacement amplification at a lever ratio of 3.6.

In recent years, significant progress has been achieved in applying piezoelectric actuators within micro-optical cavity systems. Piezoelectric thin-film actuators, characterised by their low power consumption and high responsiveness, provide an effective solution for miniaturised integrated lasers [31,32,33,34,35,36,37]. Incorporating piezoelectric dielectric cladding layers into optical waveguides can significantly reduce optical transmission losses while maintaining CMOS process compatibility, thereby laying the groundwork for high-speed tunable optical couplers and switches [38,39,40,41,42,43]. Furthermore, the use of piezoelectric actuators is expanding into areas such as acousto-optic modulation and programmable optical routing [39,44,45,46,47,48].

Nevertheless, research on the application of piezoelectric actuators in MOEMS remains relatively limited, and MOEMS devices face challenges in achieving high precision, including demands for ultra-high manufacturing accuracy and the inherent trade-off between precision and dynamic range [49]. This study proposes an innovative piezoelectric thin-film actuator tailored for micro-optical cavities. By optimizing the layout of materials such as PZT and SiO_2_ and configuring the electrodes to form an island-circular structure, combined with particle swarm optimization (PSO) to enhance displacement output capability and anti-gravity performance, this structure achieves nanometer-level displacement output and nanometer-level resolution control within a confined space. It also demonstrates potential for large-scale batch manufacturing compatible with microcavity processes. This actuator not only relaxes process tolerances during cavity manufacturing but also enables real-time cavity-length tuning, thereby improving dynamic response and signal-modulation capability—providing a key technological foundation for the development of high-performance optical MEMS sensors.

## 2. Simulation Design and Optimization

### 2.1. Design and Performance Simulation of Piezoelectric Actuators

**Figure 1:**
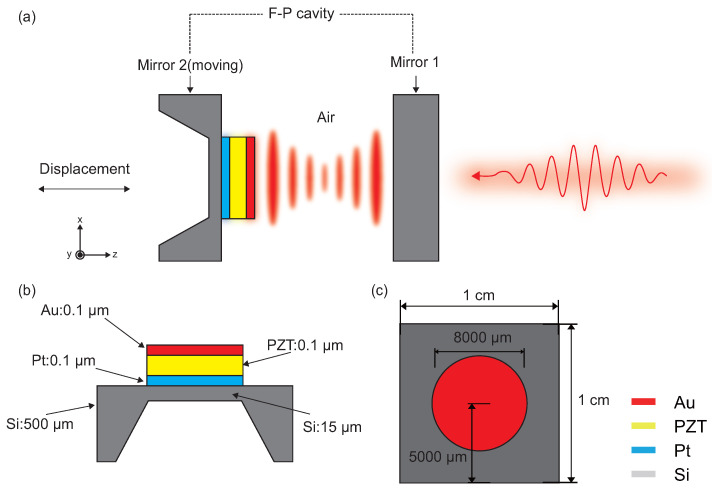
(**a**) Schematic diagram of the F-P cavity based sensor on a piezoelectric thin-film actuator. (**b**) Side view of a piezoelectric thin-film actuator (Au and Pt serve as the top and bottom electrodes). (**c**) Top view of a piezoelectric thin-film actuator structure.

As shown in Figure 1a, the studied cavity is an F-P cavity composed of two parallel mirrors. Mirror 1 serves as the “fixed” mirror, while Mirror 2 (the “moving” mirror) is attached to the object under test. We designed a piezoelectric thin-film actuator on Mirror 2 to enable active modulation of the optical cavity. As depicted in Figure 1b,c, the overall structure of this actuator features an island-circular configuration. A silicon substrate acts as the support layer, while the piezoelectric functional layer consists of lead zirconate titanate ceramic (PZT-5H). During analysis, key geometric parameters, such as the diameter of the planar electrode and the thickness of the back-etched silicon film, can be adjusted based on specific application requirements to optimize device performance.

Finite element analysis (FEA) was performed using ANSYS Mechanical 2023 R1 APDL to evaluate the structural displacement output under various driving voltages and gravitational loads. The electromechanical coupling of the piezoelectric layer was modeled using the standard constitutive equations in stress-charge form:

(1) $$\left\{\begin{array}{c} \left\{T\right\}=\left[c^{E}\right]\left\{S\right\}-{\left[e\right]}^{T}\left\{E\right\}\\ \left\{D\right\}=\left[e\right]\left\{S\right\}+\left[\varepsilon^{S}\right]\left\{E\right\} \end{array}\right.$$

where {T}, {S}, {E}, and {D} represent the stress vector, strain vector, electric field, and electric displacement, respectively; $\left[c^{E}\right]$ is the elastic stiffness matrix evaluated at constant electric field, [e] is the piezoelectric stress matrix, and $\left[\varepsilon^{S}\right]$ is the dielectric permittivity matrix evaluated at constant strain. The simulation incorporated detailed material parameters for each constituent layer, as summarized in Table 1. To accommodate the geometric complexity, the model employed an unstructured mesh of high-order tetrahedral elements—specifically, SOLID226 for the piezoelectric layer and SOLID187 for the remaining components—with convergence validated through a systematic mesh refinement study. For gravity-only analyses, the gravitational load was applied as a body force ($g=9.8\;{m/s}^{2}$) in the negative Z-direction.

**Figure 2:**
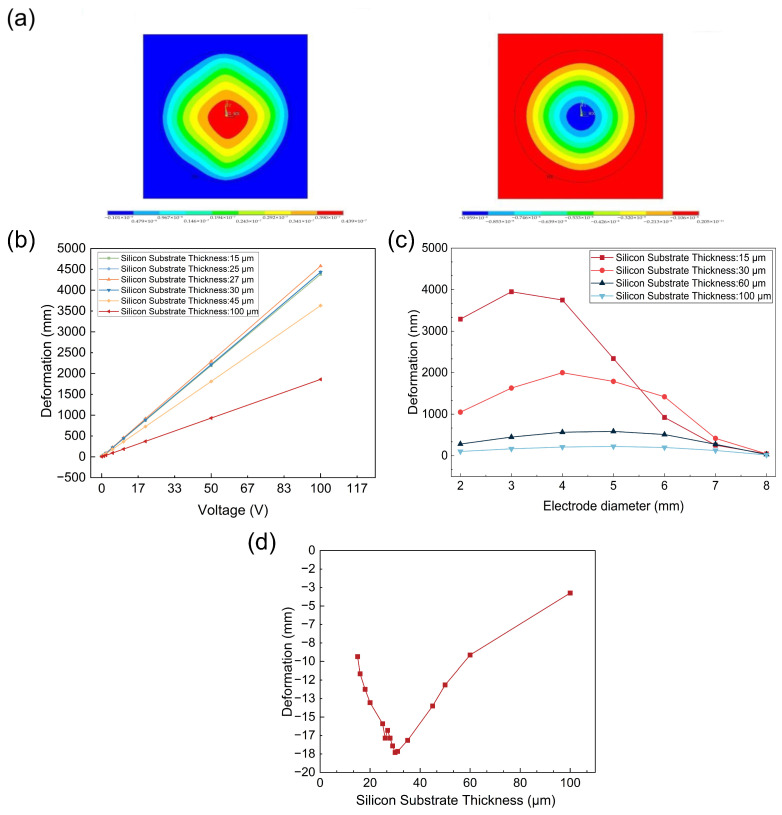
(**a**) Comparison of deformations induced by a 1 V voltage and gravity independently. (**b**) Maximum central deformation as a function of voltage for various substrate thicknesses. (**c**) Impact of electrode size on maximum central deformation at 1 V across different substrate thicknesses. (**d**) Maximum central deformation under gravity for varying substrate thicknesses.

As illustrated in Figure 2a, the activated device exhibits a highly radially symmetric strain distribution characterized by concentric rings, with deformation magnitude peaking at the geometric center. Notably, the maximum displacement demonstrates a near-linear correlation with the applied voltage. Figure 2b reveals that the displacement output remains relatively stable across a specific range of silicon substrate thicknesses before undergoing a rapid decline. Furthermore, Figure 2c shows a non-monotonic relationship between displacement and electrode diameter; as the diameter is reduced from 8 mm to 2 mm, the displacement initially increases before decreasing, identifying an optimal electrode geometry.

Finally, Figure 2d identifies the critical silicon thickness at which the device achieves peak gravitational sensitivity. These findings suggest that displacement performance can be precisely tuned by optimizing substrate thickness and electrode dimensions. Specifically, employing a moderately thicker silicon film serves as an effective strategy to mitigate environmental gravitational interference.

**Figure 3:**
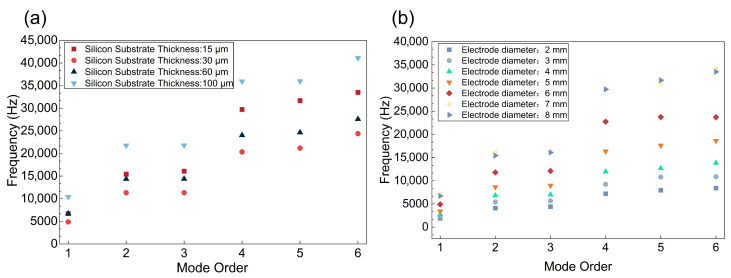
(**a**) The first six mode frequencies as a function of substrate thickness. (**b**) The first six mode frequencies as a function of electrode dimensions.

To achieve high-precision displacement output and prevent potential resonance and mechanical fatigue during operation, a modal analysis was conducted on the designed island-circular actuator to identify its natural frequency characteristics. Figure 3 shows that the first-order natural frequency of this initial parametric design model is about 6.7 kHz. It is also evident that as the substrate thickness increases, the natural frequency first decreases and then increases. Furthermore, increasing the electrode diameter enhances overall stiffness, which raises the natural frequency. In summary, to ensure stable displacement output, extend the device’s lifespan, and attain the desired excitation characteristics, the design should maximise the frequency gap between the first-order and higher-order modes while keeping the operating frequency well away from the higher-order modes.

**Figure 4:**
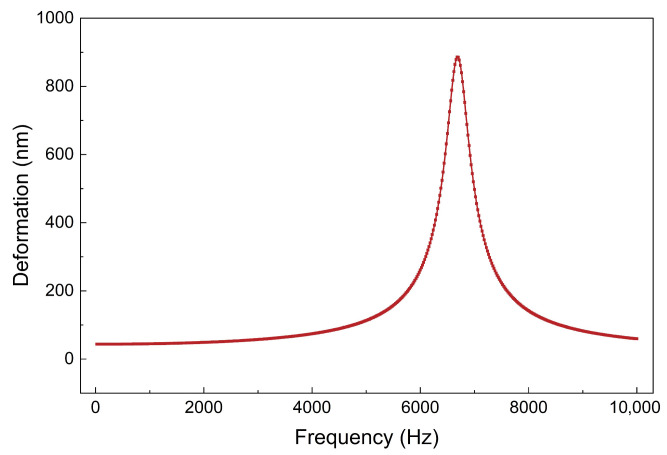
Frequency response curve of the piezoelectric thin-film actuator.

Harmonic response analysis was subsequently performed to further verify the conclusions of the modal analysis and the appropriateness of the model setup, as shown in Figure 4. When the scanning frequency approaches 6.7 kHz, the output response sharply increases and forms a peak, indicating structural resonance. This result matches the first-order natural frequency obtained from the modal analysis, corroborating the validity of the simulation model and boundary condition parameters.

### 2.2. Structural Parameter Optimization

Considering the target application scenarios and subsequent processing feasibility, this study conducts an iterative optimization design for the thin-film actuator. To enhance the actuator’s operational stability, its resistance to gravitational disturbances must be improved to suppress environmentally induced operational errors. Simultaneously, enhancing the displacement flatness expands the effective working range within precision structures, such as micro-optical cavities, thereby increasing planar utilization efficiency. Based on these performance requirements, a structural parameter optimization problem is formulated: constrained by maintaining the displacement output capability (displacement-to-voltage ratio) within the range of 200–400 nm/V, the optimization targets gravity resistance and displacement flatness. The selected design variables are the thickness of the silicon device layer and the dimensions of the electrode plane. The particle swarm optimization (PSO) algorithm is employed to solve this optimization problem and perform the iterative design [50].

To quantify the aforementioned optimization objectives, the following metrics are employed: displacement output capability is measured by the displacement magnitude of the actuator under a 1 V excitation (unit: nm/V); anti-gravity performance is assessed by the maximum sagging distance $y_{1}=f$ of the thin-film actuator when its bottom substrate is fixed and subjected solely to gravitational force. Displacement flatness is defined as the ratio of the maximum gravitational sag distance *f* to the corresponding planar electrode size *x*, that is, $y_{2}=f/x$.

Based on the above metrics, the objective function in this optimization can be expressed as:

(2) $$F=c_{1}y_{1}^{\prime }+c_{2}y_{2}^{\prime }$$

where $y_{1}$ and $y_{2}$ denote the raw optimization targets, representing the maximum gravity-induced deflection *f* and the displacement flatness f/x, respectively. To achieve optimal structural stability, both $y_{1}$ and $y_{2}$ are desired to be as small as possible; therefore, the ultimate target of the overall objective function *F* is minimization. Because $y_{1}$ and $y_{2}$ exhibit severe scale differences, Min-Max normalization is applied prior to weighting to eliminate dimensional discrepancies. The normalized values $y_{1}^{\prime }$ and $y_{2}^{\prime }$ are calculated as:

(3) $$x^{\prime }=\frac{x-\min(x)}{\max(x)-\min(x)}$$

where *x* denotes the original data and $x^{\prime }$ denotes the normalized data. The estimated bounds utilized for normalization are [1 nm, 100 nm] for $y_{1}$ and [$0.25\times 10^{-6}$, $100\times 10^{-6}$] for $y_{2}$, which are dynamically estimated based on the design space. Since both anti-gravity capability and planar flatness are equally critical for the target microcavity application, equal weights are assigned: $c_{1}=0.5$ and $c_{2}=0.5$.

The optimization process relies on real-time ANSYS finite element simulations to evaluate the fitness values, which constitutes a computationally expensive optimization problem (each evaluation requires approximately 1 min). Consequently, the convergence criterion is set to a fixed maximum number of iterations. The specific PSO parameters are configured as follows: a swarm size of 20 particles, a maximum of 20 iterations, an inertia weight (ω) linearly decreasing from 0.9 to 0.4 to effectively balance global exploration and local exploitation, and learning factors $c_{pso1}=2.0$ (cognitive) and $c_{pso2}=2.0$ (social).

The variable parameters involved in the optimization process are listed in Table 2, while all other structural dimensions remain unchanged from the initial design specified in Section 2.1. The final optimized structural performance parameters are presented in Table 3. Notably, the displacement output capability of the optimized actuator is 207.6 nm/V, securely falling within the preset constraint range of 200–400 nm/V. Furthermore, the anti-gravity performance is significantly enhanced (with the sagging distance reduced from 10.4 nm to 3.8 nm), and the displacement flatness is correspondingly improved (reduced from 2.6 × 10^−6^ m/m to 1.3 × 10^−6^ m/m).

## 3. Results and Discussion

### 3.1. Fabrication of Piezoelectric Thin-Film Actuators

**Figure 5:**
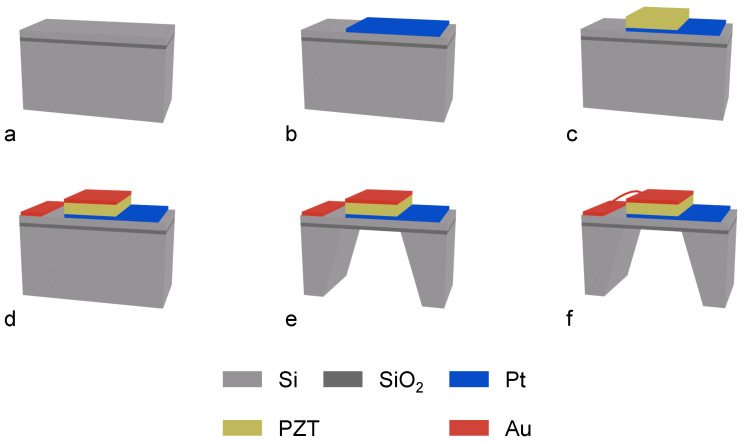
Schematic illustration of the fabrication process flow for the piezoelectric thin-film actuator. (**a**) SOI substrate. (**b**) Deposition and patterning of the bottom electrode and right-side bonding pad. (**c**) Deposition and patterning of the PZT piezoelectric layer. (**d**) Deposition and patterning of the top electrode and left-side bonding pad. (**e**) Deep silicon dry etching down to the buried oxide layer, leaving a residual silicon thickness of approximately 15 µm. (**f**) Wire bonding process connecting the top electrode to the left-side bonding pad.

Figure 5 illustrates the fabrication process flow for MEMS-based piezoelectric thin-film actuators. The process utilizes a 4-inch SOI wafer as the substrate, featuring a buried oxide layer thickness of 1–2 µm and a top-layer single-crystal silicon thickness of approximately 15 µm.

First, a 100 nm thick platinum (Pt) bottom electrode was deposited onto the top-layer monocrystalline silicon surface via magnetron sputtering. Subsequently, a 2 µm thick lead zirconate titanate (PZT) piezoelectric film was prepared on the bottom electrode using magnetron sputtering. The planar dimensions of the PZT layer were controlled via wet etching to be 3–20 µm larger than the bottom electrode, ensuring electrical isolation between the top and bottom electrodes.

Next, a 100 nm thick gold (Au) electrode was sputter-deposited onto the surface of the PZT layer. Its planar dimensions were designed to be 60–80% of the lower electrode to prevent short circuits between the upper and lower electrodes. Subsequently, a portion of the silicon substrate was removed from the back side of the wafer using deep reactive ion etching (DRIE) until the buried oxide layer halted the process, keeping the actuator structure layer thickness at approximately 15 µm. Finally, the electrode on the PZT layer was electrically connected to the pad on the left side of the device via wire bonding.

**Figure 6:**
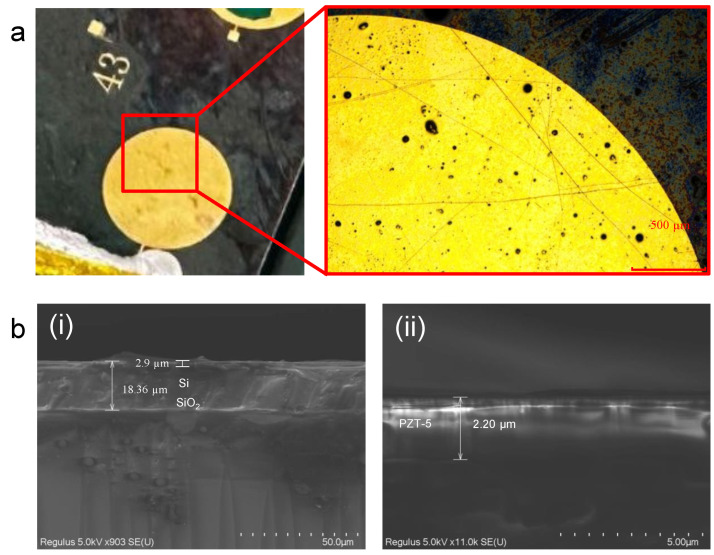
Photograph of the fabricated piezoelectric thin-film actuator. (**a**) Optical micrograph showing the surface view. (**b**) Scanning electron micrograph (SEM) of (**i**) the cross-sectional view of the actuator and (**ii**) the thickness of the PZT film.

Figure 6 shows the completed piezoelectric thin-film actuator sample. The PZT layer and metal electrode structure exhibit integrity and good morphology. It should be noted that the total thickness observed in cross-sectional SEM images includes not only the PZT layer but also the underlying adhesion and electrode layers between PZT and the Si substrate. Therefore, minor variations from the nominal PZT thickness can be attributed to these additional layers, as well as to typical measurement uncertainties and process tolerances. Furthermore, the white material visible near the electrode in Figure 6a is conductive silver paste, which was applied during the fabrication process to ensure a robust electrical connection. The liquid black spots visible on the metal film surface are residual traces of this conductive paste, while the black fine lines are scratches caused by the movement of the etching pad.

### 3.2. Experimental Performance Comparison and Analysis

**Figure 7:**
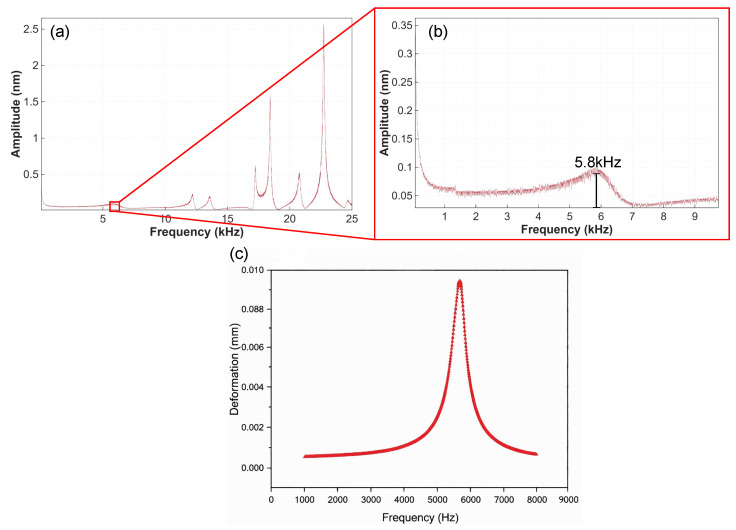
(**a**) Approximate amplitude-frequency response curve of the structure within the 0 to 25 kHz range. (**b**) Enlarged view of (**a**) within the 0 to 10 kHz range. (**c**) Measured frequency response curve of the experimental prototype.

To systematically evaluate the performance of the piezoelectric thin-film actuator and compare it with simulation results, its first-order natural frequency was first analyzed, as shown in Figure 7. Based on the physical system testing, the measured first-order natural frequency is approximately 5.8 kHz. Meanwhile, the updated simulation result for the fabricated device is approximately 5.66 kHz (Figure 7c), exhibiting a relative error of only 2.4%, which demonstrates good agreement. Additionally, discrepancies exist in the response amplitude. Preliminary analysis suggests these differences may stem from multiple factors: variations in materials used during actual fabrication (e.g., PZT type), structural dimensional deviations, environmental damping, inconsistencies between LDV laser measurement point locations and simulation settings, as well as discrepancies between the actual output of the signal generator during sweep testing and the ideal excitation conditions simulated.

**Figure 8:**
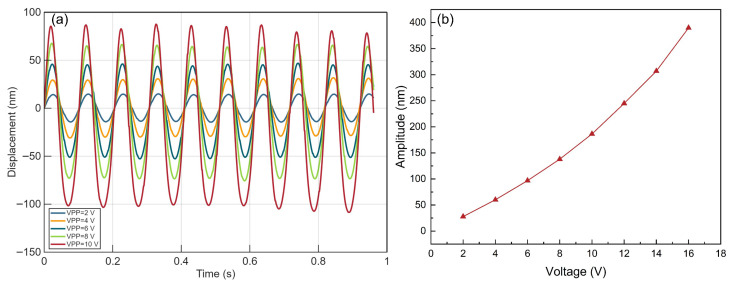
(**a**) Response of the piezoelectric thin-film actuator under sinusoidal excitation at different voltage amplitudes (1 kHz). (**b**) Relationship between response amplitude and applied voltage magnitude at 1 kHz.

To further investigate the displacement output characteristics of the actuator under AC signal excitation, its response was tested with sinusoidal voltages of different amplitudes, and the results are shown in Figure 8. The experiment employed standard sinusoidal signals at a frequency of 1 kHz with peak-to-peak voltages ($V_{pp}$) of 2 V, 4 V, 6 V, 8 V, 10 V, 12 V, 14 V, and 16 V as excitation. Figure 8a displays the response waveforms and their amplitudes for the first five voltage levels. Figure 8b shows that the response amplitude increases with higher excitation voltages, though not in a strictly linear way, but rather with an accelerated growth trend. This can be explained by the increasingly significant contribution of kinetic energy to deformation during intense oscillation.

**Figure 9:**
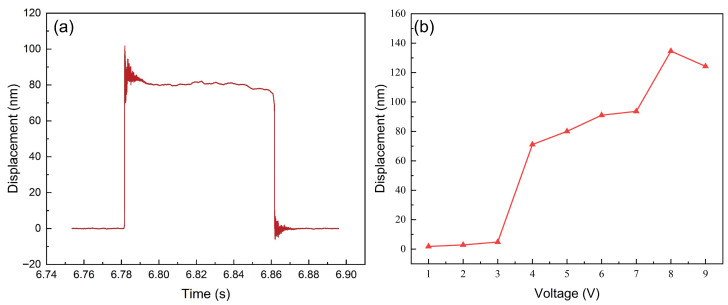
(**a**) A dynamic response from initial excitation to recovery at 5 V. (**b**) Steady-state response amplitude versus applied voltage curve.

Throughout the entire experiment, the LDV laser measurement point remained fixed at the geometric center of the actuator. Figure 9a shows the displacement-time curve of the piezoelectric film actuator during the entire process from deformation to recovery under excitation by a pulse signal with a frequency of 5 Hz (period 200 ms), a duty cycle of 40%, a rise/fall time of 10 ns, a high level of 5 V, and a low level of 0 V. It is evident that the actuator exhibits oscillation accompanied by free-running decay at both the onset and removal of voltage application. During the voltage maintenance phase, the displacement remains stable. Under these excitation conditions, the actuator’s displacement output capability is 16.736 nm/V. Compared to the 15.575 nm/V measured under 5 Vpp AC excitation, the difference is only 6.937%, indicating good consistency between the two excitation methods under identical boundary conditions and high experimental reliability.

To further examine the effect of voltage amplitude on response, a steady-state voltage excitation ranging from 1 V to 9 V was applied to the actuator, with the extended results shown in Figure 9b. The experiment revealed distinct performance regimes. The actuator displayed optimal linear response characteristics within the 4 V to 6 V range; in this optimal window, the displacement increased steadily from 71.11 nm to 91.00 nm, exhibiting excellent linearity ($R^{2}=0.997$) and a stable average displacement sensitivity of approximately 9.95 nm/V. When the voltage dropped below 4 V, deformation decreased sharply, resulting in a “disengagement” phenomenon. Conversely, when the voltage exceeded 6 V, the actuator entered a degraded non-linear range where linearity dropped severely ($R^{2}=0.691$ in the 6–9 V range). In this higher-voltage regime, the step-by-step sensitivity exhibited extreme variance: the local sensitivity plummeted to just 2.66 nm/V between 6 V and 7 V, spiked erratically to 40.92 nm/V between 7 V and 8 V, and then entirely reversed to a negative sensitivity of −10.31 nm/V (with displacement dropping to 124.27 nm) at 9 V. This massive variance in local sensitivity and the non-monotonic displacement behavior quantitatively confirm that the piezoelectric torque generated by the inverse piezoelectric effect was nearing its limit, resulting in “oscillatory” vibrations and poor repeatability. Therefore, to ensure the stability and linearity of the output under steady-state excitation, the actuator must be calibrated to the actual operating conditions, and the excitation voltage range should be strictly controlled within its optimal window.

### 3.3. Experimental Verification of Dynamic Tuning Performance in Micro-Optical Cavities

**Figure 10:**
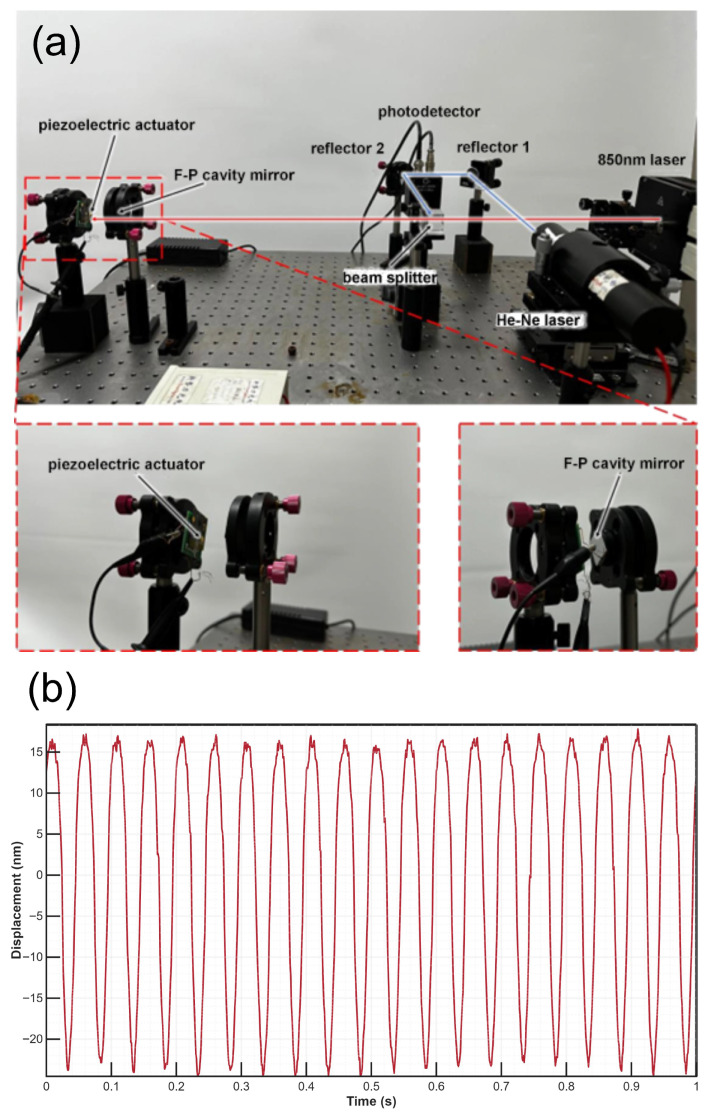
(**a**) Experimental configuration of piezoelectric actuators based on F-P cavity performance measurement. (**b**) Displacement response under sinusoidal excitation at 2 Vpp.

This study presents the experimental setup shown in Figure 10a to validate the real-world performance of the designed piezoelectric thin-film actuator within an F-P cavity. The actuator functions as the movable mirror inside the F-P cavity, while an 850 nm wavelength laser serves as the probing light. By dynamically adjusting the cavity length through actuation, the modulation response of the transmitted optical intensity is experimentally assessed to evaluate the actuator’s dynamic performance in real-time cavity control.

Figure 10b presents the displacement response of the piezoelectric thin-film actuator during dynamic operation. To accurately reflect the dynamic tuning performance, these displacement results were derived directly from the raw optical cavity response. Specifically, the dynamic bending of the actuator modulates the F-P cavity length, which induces a phase shift in the resonating light. This phase shift is subsequently detected by a photodetector as variations in light intensity and recorded as corresponding voltage signals. By operating the system within the linear region of the voltage-displacement relationship, these raw optical signals are converted into real-time physical displacement. The conversion is calibrated based on the optical fringe peak-to-peak voltage, which corresponds to the half-wavelength (λ/2=425 nm) of the 850 nm testing laser.

A certain deviation is observed between the measured intensity modulation and the displacement response recorded by a laser Doppler vibrometer (LDV). This discrepancy may be caused by several factors: minor surface irregularities or contamination on the actuator resulting in optical scattering; beam spreading due to the top titanium metal layer; and cumulative systematic errors from manual alignment and measurement processes. Despite these quantitative differences, the results demonstrate the feasibility of using the piezoelectric thin-film actuator as a movable mirror in an F-P cavity. By modulating the actuator’s bending deformation, the optical path length within the cavity can be actively controlled, enabling dynamic modulation of both intensity and phase. This method not only provides excellent tunability and rapid response but also improves the robustness of micro-optical MEMS sensors against fabrication tolerances. It allows large-range, high-precision active tuning and phase modulation, greatly expanding the application potential of MOEMS in sensing, signal modulation, and integrated photonic devices.

**Figure 11:**
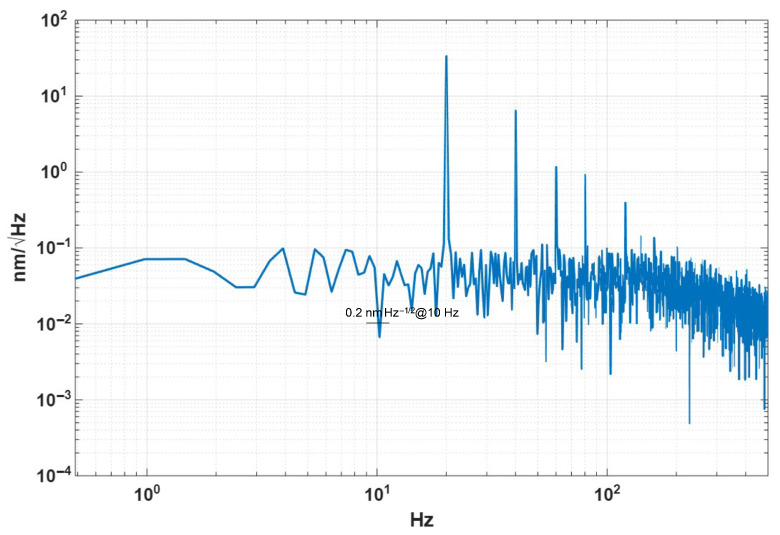
Power spectral density of the displacement noise.

Figure 11 displays the displacement noise density spectrum across the frequency range from 0.5 Hz to 400 Hz. The figure reveals a noise floor of approximately $10^{-2}\;\mathrm{nm}/\sqrt{\mathrm{Hz}}$ near 10 Hz. Significant narrowband peaks appear at 20 Hz and its integer multiples, attributed to vibrations induced by piezoelectric actuation.

## 4. Conclusions

This paper presents the design, fabrication, and experimental validation of a piezoelectric thin-film actuator tailored for the dynamic control of micro-optical cavities. Adopting an island-circular multilayer thin-film structure based on the $d_{33}$ mode piezoelectric effect, the actuator demonstrates reasonable output linearity, robust interference resistance, and high process compatibility. By coupling finite element simulations with particle swarm optimization (PSO) algorithms, the device is engineered to deliver nanometer-scale displacement and high-resolution control within a confined micro-scale footprint, significantly enhancing the adjustability and environmental adaptability of microcavity reflective surfaces. Experimental evaluations reveal a first-order natural frequency of approximately 5.8 kHz, alongside a consistent displacement response under a 1 kHz AC excitation. Furthermore, the actuator exhibits stable linear characteristics within an optimal steady-state drive voltage range of 4–6 V, successfully validating its integration feasibility within a Fabry-Pérot cavity system. Ultimately, this study provides an effective strategy for relaxing MOEMS fabrication tolerances and enabling high-precision optical phase modulation, laying a crucial foundation for the development of high-performance micro-optical sensing systems. Future work will focus on optimizing the heterogeneous integration processes between piezoelectric actuators and microcavities, advancing multi-physics coupling models, and verifying practical applications in broadband dynamic modulation systems.

## Figures and Tables

**Table 1:** Material properties of the simulated structure. properties of the simulated structure.

Property	Si	Pt	PZT-5H	Au
Elastic Modulus (GPa)	$E_{\mathrm{Si}}=112$	$E_{\mathrm{Pt}}=160$	$E_{\mathrm{PZT}}=60$	$E_{\mathrm{Au}}=79$
Poisson’s ratio	$\nu_{\mathrm{Si}}=0.25$	$\nu_{\mathrm{Pt}}=0.39$	$\nu_{\mathrm{PZT}}=0.29$	$\nu_{\mathrm{Au}}=0.44$
Density (kg/m^3^)	$\rho_{\mathrm{Si}}=2328$	$\rho_{\mathrm{Pt}}$ = 21,450	$\rho_{\mathrm{PZT}}=7500$	$\rho_{\mathrm{Au}}$ = 19,320
$d_{31}$ (pC/N)	-	-	−274	-

**Table 2:** Optimized Dimensional Parameters of Island-circular Actuator.

Parameter 1	Upper Limit	Lower Limit	Parameter 2	Upper Limit	Lower Limit
Substrate Thickness	100 µm	15 µm	electrode diameter	8 mm	2 mm

**Table 3:** Optimized Performance Metrics of the Island-circular Actuator.

Gravitational Resistance Capability		Displacement Output Uniformity		Displacement Output Capability	
Before	After	Before	After	Before	After
10.4 nm	3.8 nm	$2.6\times 10^{-6}$ m/m	$1.3\times 10^{-6}$ m/m	46.9 nm/V	207.6 nm/V

## Data Availability

The raw data supporting the conclusions of this article will be made available by the authors on request.

## References

[1] Qu Z., Ouyang H., Liu H., Hu C., Tu L.C., Zhou Z. (2022). 2.4 ng/$\sqrt{\mathrm{Hz}}$ low-noise fiber-optic MEMS seismic accelerometer. Opt. Lett..

[2] Zhao M., Jiang K., Bai H., Wang H., Wei X. (2020). A MEMS based Fabry–Pérot accelerometer with high resolution. Microsyst. Technol..

[3] Du Y., Zou C.L., Zhang C., Wang K., Qiao C., Yao J., Zhao Y.S. (2020). Tuneable red, green, and blue single-mode lasing in heterogeneously coupled organic spherical microcavities. Light Sci. Appl..

[4] Hao X., Zhao S., Gao J., Suo L. (2023). All-optical tunable whispering gallery mode lasing in a PMMA-coated microcavity embedded with a high-efficiency nanoheater. Opt. Laser Technol..

[5] Zou H., Shi Y. (2024). A polarization sensitive self-excited multi-wavelength Brillouin-Erbium fiber laser without filtering devices. Opt. Laser Technol..

[6] Corcoran B., Mitchell A., Morandotti R., Oxenløwe L.K., Moss D.J. (2025). Optical microcombs for ultrahigh-bandwidth communications. Nat. Photonics.

[7] Powell K., Li L., Shams-Ansari A., Wang J., Meng D., Sinclair N., Deng J., Lončar M., Yi X. (2022). Integrated silicon carbide electro-optic modulator. Nat. Commun..

[8] Lu Q., Xiao Q., Liu C., Wang Y., Zhu Q., Xu M., Wang X., Wang X., Huang W. (2023). Inverse design and realization of an optical cavity-based displacement transducer with arbitrary responses. Opto-Electron. Adv..

[9] Xiao Q., Wu S., Wang Y., Liu C., Feng W., Yao Y., Huang P., Wang X., Lu Q. (2023). Error analysis and realization of a phase-modulated diffraction grating used as a displacement sensor. Opt. Express.

[10] Guo Y., Liang Y., Li Y., Tian B., Fan X., He Y., Liu M., Peng L., Tang N., Tan T. (2024). Optical Microcavities Empowered Biochemical Sensing: Status and Prospects. Adv. Devices Instrum..

[11] Loyez M., Adolphson M., Liao J., Yang L. (2023). From Whispering Gallery Mode Resonators to Biochemical Sensors. ACS Sens..

[12] van Haagen S., Nur S., Ishihara R. (2025). Deep Learning-Optimized, Fabrication Error-Tolerant Photonic Crystal Nanobeam Cavities for Scalable On-Chip Diamond Quantum Systems. ACS Appl. Opt. Mater..

[13] Maier P., Rupp S., Lettner N., Hecker Denschlag J., Kubanek A. (2025). Fabrication of customized low-loss optical resonators by combination of FIB-milling and CO_2_ laser smoothing. Opt. Express.

[14] Javid U.A., Rogers S.D., Graf A., Lin Q. (2021). Cavity Optomechanical Sensing in the Nonlinear Saturation Limit. Laser Photonics Rev..

[15] Tang J., Liu J., Shang C., Xie C., Guo H., Qian K., Xue C., Liu J. (2015). Fabrication and spectral characterizations of high Q asymmetric resonant cavities. Opt. Commun..

[16] Peixoto R., Pires J.S., Monteiro C.S., Raposo M., Ribeiro P.A., Silva S.O., Frazão O., Lopes J.V.P. (2021). Environmental Sensitivity of Fabry-Perot Microcavities Induced by Layered Graphene-Dielectric Hybrid Coatings. Phys. Rev. Appl..

[17] Zhi Y., Yu X.C., Chen H.J., Guan B.O., Xiao Y.F. (2019). Noise suppression of mechanical oscillations in a microcavity for ultrasensitive detection. Opt. Lett..

[18] Saavedra C., Pandey D., Alt W., Pfeifer H., Meschede D. (2021). Tunable fiber Fabry-Perot cavities with high passive stability. Opt. Express.

[19] Bulgan E., Kanamori Y., Hane K. (2008). Submicron silicon waveguide optical switch driven by microelectromechanical actuator. Appl. Phys. Lett..

[20] Han S., Seok T.J., Quack N., Yoo B.W., Wu M.C. (2015). Large-scale silicon photonic switches with movable directional couplers. Optica.

[21] Lee M.C., Wu M. (2005). MEMS-actuated microdisk resonators with variable power coupling ratios. IEEE Photonics Technol. Lett..

[22] Rabih A.A.S., Nabavi S., Ménard M., Nabki F. (2024). Multi Degrees-of-Freedom Hybrid Piezoelectric-Electrostatic MEMS Actuators Integrated With Displacement Sensors. J. Microelectromech. Syst..

[23] Zhou X., Wu S., Wang X., Wang Z., Zhu Q., Sun J., Huang P., Wang X., Huang W., Lu Q. (2024). Review on Piezoelectric Actuators: Materials, Classifications, Applications, and Recent Trends. Front. Mech. Eng..

[24] Mangi M.A., Elahi H., Ali A., Jabbar H., Aqeel A.B., Farrukh A., Bibi S., Altabey W.A., Kouritem S.A., Noori M. (2025). Applications of piezoelectric-based sensors, actuators, and energy harvesters. Sens. Actuators Rep..

[25] Chang Q., Chen W., Zhang S., Deng J., Liu Y. (2024). Review on Multiple-Degree-of-Freedom Cross-Scale Piezoelectric Actuation Technology. Adv. Intell. Syst..

[26] Heo S., Wiguna T., Park H.C., Goo N.S. (2007). Effect of an Artificial Caudal Fin on the Performance of a Biomimetic Fish Robot Propelled by Piezoelectric Actuators. J. Bionic Eng..

[27] Hall A.J., Riddick J.C. (2012). Micro-electro-mechanical flapping wing technology for micro air vehicles. Proceedings of the Bioinspiration, Biomimetics, and Bioreplication 2012.

[28] Sun X., Chen W., Tian Y., Fatikow S., Zhou R., Zhang J., Mikczinski M. (2013). A Novel Flexure-Based Microgripper with Double Amplification Mechanisms for Micro/Nano Manipulation. Rev. Sci. Instrum..

[29] Hua S.M., Cheng G.m., Zhang Z., Zeng P. (2010). Precise Impact Drive Mechanism Based on Asymmetrically Clamped Piezoelectric Actuator. Appl. Mech. Mater..

[30] Kim Y.W., Choi S.C., Park J.W., Jung Y.H., Lee D.W. (2008). The characteristics of variable speed inchworm stage using lever mechanism by different materials. J. Nanosci. Nanotechnol..

[31] Siddharth A., Attanasio A., Bianconi S., Lihachev G., Zhang J., Qiu Z., Bancora A., Kenning S., Wang R.N., Voloshin A.S. (2024). Piezoelectrically tunable, narrow linewidth photonic integrated extended-DBR lasers. Optica.

[32] Dai T., Wang Y., Wu X., Wu J., Yao B., Ju Y., Shen Y. (2018). An injection-seeded Q-switched Ho: YLF laser by a tunable single-longitudinal-mode Tm, Ho: YLF laser at 2050.96 nm. Opt. Laser Technol..

[33] Li G., Li Y., Yang K., Liu M. (2013). A linearity tunable DBR fiber laser based on closed-loop PZT. Opt. Laser Technol..

[34] Lihachev G., Riemensberger J., Weng W., Liu J., Tian H., Siddharth A., Snigirev V., Shadymov V., Voloshin A., Wang R.N. (2022). Low-Noise Frequency-Agile Photonic Integrated Lasers for Coherent Ranging. Nat. Commun..

[35] Lihachev G., Bancora A., Snigirev V., Tian H., Riemensberger J., Shadymov V., Siddharth A., Attanasio A., Wang R.N., Visani D. (2023). Frequency agile photonic integrated external cavity laser. (CLEO). APL Photonics.

[36] Buric M., Falk J., Chen K.P., Cashdollar L., Elyamani A. (2006). Piezo-electric tunable fiber Bragg grating diode laser for chemical sensing using wavelength modulation spectroscopy. Opt. Express.

[37] Liu J., Tian H., Lucas E., Raja A.S., Lihachev G., Wang R.N., He J., Liu T., Anderson M.H., Weng W. (2020). Monolithic Piezoelectric Control of Soliton Microcombs. Nature.

[38] Dong B., Tian H., Zervas M., Kippenberg T.J., Bhave S.A. PORT: A piezoelectric optical resonance tuner. Proceedings of the 2018 IEEE Micro Electro Mechanical Systems (MEMS).

[39] Tian H., Liu J., Attanasio A., Siddharth A., Blésin T., Wang R.N., Voloshin A., Lihachev G., Riemensberger J., Kenning S.E. (2024). Piezoelectric actuation for integrated photonics. Adv. Opt. Photonics.

[40] Everhardt A., Tran T.L.A., Mitsolidou C., Horner T.R., Grootjans R., Oldenbeuving R., Heuvink R., Geuzebroek D., Leinse A., Roeloffzen C. (2022). Ultra-low power stress-based phase actuation in TriPleX photonic circuits. Proceedings of the Integrated Optics: Devices, Materials, and Technologies XXVI.

[41] Jin W., Polcawich R.G., Morton P.A., Bowers J.E. (2018). Piezoelectrically tuned silicon nitride ring resonator. Opt. Express.

[42] Jiang W., Mayor F.M., Patel R.N., McKenna T.P., Sarabalis C.J., Safavi-Naeini A.H. (2020). Nanobenders as Efficient Piezoelectric Actuators for Widely Tunable Nanophotonics at CMOS-level Voltages. Commun. Phys..

[43] Liu Y., Odedeyi T., Zervas G. (2024). Switching in microseconds: Design of a 5 × 5 non-blocking free space optical switch at 1550nm with a piezo-actuator and beam-steering lens system. Opt. Express.

[44] Dong M., Clark G., Leenheer A.J., Zimmermann M., Dominguez D., Menssen A.J., Heim D., Gilbert G., Englund D., Eichenfield M. (2022). High-Speed Programmable Photonic Circuits in a Cryogenically Compatible, Visible–near-Infrared 200 Mm CMOS Architecture. Nat. Photonics.

[45] Li H., Tadesse S.A., Liu Q., Li M. (2015). Nanophotonic cavity optomechanics with propagating acoustic waves at frequencies up to 12 GHz. Optica.

[46] Shao L., Yu M., Maity S., Sinclair N., Zheng L., Chia C., Shams-Ansari A., Wang C., Zhang M., Lai K. (2019). Microwave-to-optical conversion using lithium niobate thin-film acoustic resonators. Optica.

[47] Zhang L., Cui C., Chen P.K., Fan L. (2024). Integrated-waveguide-based acousto-optic modulation with complete optical conversion. Optica.

[48] Wan L., Huang J., Wen M., Li H., Zhou W., Yang Z., Chen Y., Liu H., Zeng S., Liu D. (2025). Hybrid Thin-Film Lithium Niobate Micro-Ring Acousto-Optic Modulator with Low Half-Wave-Voltage-Length Product. Laser Photonics Rev..

[49] Wu S., Yan W., Wang X., Xiao Q., Wang Z., Sun J., Yu X., Yang Y., Zhu Q., Yang G. (2025). A *μ*Gal MOEMS Gravimeter Designed with Free-Form Anti-Springs. Nat. Commun..

[50] Lu Q., Sun J., Wang Z., Wang C., Wang X., Wang X., Wu S., Zhou X., Sun J., Zhan Z. (2025). Adaptive elite learning particle swarm optimization algorithm with complementary sub-strategies for multimodal problems. Sci. China Inf. Sci..

